# Supplemental Review; or Quarterly Periscope of Practical Medicine, Surgery, &c.

**Published:** 1821-12-01

**Authors:** 


### Supplemental Review

AND

### QUARTERLY PERISCOPE

OF

### PRACTICAL MEDICINE, SURGERY, &c. &c.

*WITH COMMENTARIES.*

*Paucis libris immorari et innutriri oportet, si velis aliquid trahere, quod in animo fideliter hæreat.* SENECA.

Duo vitia vitanda sunt in cognitionis et scientiæ studio. ***** Alterum est vitium, quod quidam nimis magnam operam conferunt in res obscuras atque difficiles, easdemque non necessarias. CICERO.

OUR Periscope becomes every day more necessary. The press, in every country, now groans with periodical publications, while not one paper in ten, communicated through them, is worth the perusal. A principle of *selection* is not only necessary but a capacity of *discrimination* also; for what are put forth as *interesting facts* are sometimes, we fear, but one degree removed above *ingenious fictions!* Even

*truth* comes to us so overwhelmed with words, and *facts* so diluted with ideas, that the labour of picking the pearls of knowledge out of the dunghill of literature is a most tiresome and difficult office. We shall not therefore spread a galaxy of materials before our readers more calculated to dazzle their imaginations than strengthen their judgment; —on the contrary, we shall exercise the utmost discrimination which time and experience have furnished us with, in hopes that our vigils may be useful to our junior brethren at least—while to the sages of the profession we appeal for the correctness of the line which we endeavour to draw between insignificance and worth.

It may be proper, however, to state, that the preceding remarks apply almost exclusively to the foreign journals, and that we shall very seldom notice those papers circulated by our own periodicals—more especially those in our most respected cotemporary of Edinburgh; because the latter journal has a range equal, if not superior, to our own, and is always freighted with valuable merchandize. When journals travel in the same channels, it is unnecessary for one to notice the productions of the other, unless for the sake of some practical comment or illustration. It is almost needless to state that these brief analytical sketches are confined to original articles. To review reviews would be to sift water, and gravely present to the public what we caught in the sieve.

1. *Contusions on the Epigastrium.** We are all familiar, from our school-boy days, with the distressing sensations produced by even a slight blow on the epigastrium. The following cases will be found interesting, not only as to the nature of the injury, but as to the remedial measures pursued in one of them.

*Case* 1. A soldier in the 1st regiment of Curassiers, 29 years of age, received a blow, by the kick of a horse, on the epigastrium, 18th October last, which felled him to the ground, whence he could not rise. He was bled instanter, and conveyed to the military hospital of the guards, where Baron Larrey found him motionless, pale, skin cold, eyes fixed, breathing feeble and slow, pulse scarcely perceptible. The epigastrium was so tender that the least pressure there caused the patient to utter groans. He otherwise spoke not. There was no appearance of injury on the part. Six cupping glasses (cum ferro) were applied to the epigastrium and vicinity. The application was very painful, but gave some relief to the patient, who opened his eyes, and expressed by interrupted words a sense of betterness. The pulse rose

* Revue Medicale, August 1821. M. Duponchal.

a little. This operation finished, Baron Larrey caused a sheep to be instantly pithed, and the skin, torn reeking from the body, to be enveloped round that of the patient. Round this hide again were thrown warmed blankets, in order that the temperature might be kept elevated for some time. In this situation the man remained two hours, at the expiration of which time things were greatly altered for the better. The coldness of the surface was gone—some colour returned to the face—and the patient could give an account of the accident. Nevertheless he passed a very bad night, continually complaining. On the succeeding morning he presented the following symptoms. Epigastrium and abdomen still very painful and tumefied—violent pain in the right shoulder, which induced a suspicion that the liver was injured—the pulse was full, strong, quick—tongue and mouth dry—thirst ardent;—but there was neither hiccup, vomitting, nor head-ache. The eyes were sunken, and very red—the face pale, and the features sharpened, exhibiting the expression of intense suffering. Warm bath, copious venesection, cupping, warm embrocations, diluents, no food.

The third morning presented little change. The tenderness of the abdomen was still extreme, and the other symptoms continued—and in addition the patient felt as if the right arm was fractured. On this member camphorated oil was rubbed. Emollient fomentations to the abdomen—diluent drink. On the fourth day, although the pain of the epigastrium was acute, yet the patient could bear pressure on the part somewhat better. The other symptoms were rather milder—the bowels were relaxed throughout the complaint—but the pain in the arm principally engrossed the patient's attention. He still insisted that there was a fracture there. A blister to the epigastrium. From this time the symptoms became gradually ameliorated, and, by the 12th day, convalescence was assured, and appetite restored. Convalescence continued till the 24th November, when some irregularity determined a relapse, presenting all the symptoms of acute hepatitis, which, however, gave way to local bleedings, blisters, and at last the moxa. He was discharched to duty in the beginning of December.

M. Duponchal thinks that this patient would certainly have been lost had it not been for the extraordinary measure put in force by Baron Larrey, as above related. This was the third successful employment of the same measure by the Baron, at the Military Hospital. The application of a recently excoriated sheep's hide is directed by Ambrose Paré, as a last resource in violent contusions, and where life is menaced by the first effects of the shock. Baron Larrey, in the first volume of his Memoirs and Campaigns, reports the instance of some sailors who were shipwrecked on the coast of Labrador, and picked up on the beach by the Esquimaux Indians, almost dead with cold, and exhausted with fatigue. These hospitable creatures placed the sailors on dried skins, chafed their bodies and limbs with hot aromatic liquors, and then enveloped them in the freshly excoriated hides of animals which they killed for the purpose. The inhabitants of Upper Egypt also are acquainted with this remedy. In violent, but very circumscribed contusions, they eviscerate a dove or other bird, and apply it warm to the part —but, where the contusion is extensive, they surround the body of the patient with the hide of a sheep just stripped from the body of the animal. In the memorable Russian Campaign, it is said that the most effectual mode of preventing the complete congelation of a limb, was to fold it in the skin of an animal just killed—or even to plunge the member into the reeking entrails of a horse.

We are disposed to agree with the author of the paper before us, that there is something more congenial in a warm, and we might say half-alive substance of this kind, than in any unorganized medium through which a similar degree of temperature could be kept to the part or whole of a human body. Our author proposes the application in question, in cases of repelled eruption, and to a limb after the operation for aneurism. To these propositions we can have no objection.

*Case* 2. This had not so favourable a termination as the former. On the evening of the 3d May, 1820, N——, a soldier in the Royal Guard, received, while attempting to mount a horse, so violent a kick on the umbilical region as felled him to the earth. He was instantly carried to the military hospital, and the surgeon on duty applied to the abdomen several cupping-glasses with scarificators, which abstracted an abundance of blood. This measure produced but little relief, and the patient passed the night in great anxiety. In the morning, when the surgeon in chief visited the wards, the patient was found lying on his back, the breathing difficult, the heart beating irregularly, the pulse scarcely perceptible, the extremities cold, the lower belly very painful, especially on pressure, or the least motion of the body. More blood was drawn from the abdominal parietes by cupping, and the patient was immersed in a tepid bath, after which, frictions, with camphorated oil of chamomile were employed. A calm of a few minutes was followed by the most alarming symptoms, which terminated his life about fourteen hours after the accident.

*Necrotomy.* No external mark of contusion on the abdomen; but the peritoneum reflected over the internal surface of its anterior parietes, as well as that covering the intestine, was of a deep red colour, and presenting all the phenomena of intense phlegmasia. Recent albuminous exudations had already agglutinated the convolutions of the intestines, and a pint and a half of a reddish coloured serum was effused in the abdominal cavity. The coats of the stomach were slightly injected. The duodenum appeared sound, but there was a circular hole in the jejunum, surrounded with a margin of ecchymosis. Nothing particular was observable in any other viscus of the thorax or abdomen.

The narrator has no doubt that the main cause of death was the rupture of the intestine and the extravasation thence producing peritonitis. The *immediate* cause of death, however, he attributes to *pain*, as happens in almost all the hyper-acute inflammations of serous membranes. There is nothing wonderful in the internal rupture without external mark of violence, in cases of blows on the abdomen, as we have often witnessed.

2. *Pestilence.** Dr. Hancock endeavours to steer a middle course between the contagionists and anti-contagionists; and not unreasonably expects to be "exposed to severe animadversions from those who look entirely to the wide ocean of atmospheric impurity, with boundless curiosity on one hand; as well as those who explore with confined research every creek and harbour for the *fomes* of imported contagion on the other." It will perhaps appear, Dr. Hancock thinks, that "neither the comet's glare nor the smuggler's infected bale ought to seduce our attention from the consideration of causes operating perhaps secretly and surely within the sphere of our own institutions."

"And if some, from a restless spirit of generalization, will draw conclusions, beyond a rational induction, let them have some little regard for those, who, not having joined themselves to either side, while they are anxious to discover the general laws that will embrace the contending facts and opinions, hesitate still to settle a single point which the authority of science will not sanction." *Pref.* viii.

We agree with Dr. Hancock, as far as science, or at least scientific discussions are concerned; but when legislative measures come to be acted on, the "*tutissimus in medio*" maxim is impracticable. As for instance, when a pestilential disease breaks out at Cadiz, how can the Governor of Gibraltar take half measures, and set up a scientific investigation to settle how far quarantine is necessary? He must either shut the gates or leave them wide open—for we may be pretty certain that no jury of medical men would ever come to a *unanimous* decision as to contagion or non-contagion. The Government have long seen this; and, after contemplating the everlasting wranglings, quibblings, contradictions, doubts, vacillations, and abuses, among the faculty, can we wonder at their taking the business entirely out of our hands, and leaving it to the discretion of civil or military commandants, regardless of the learned lucubrations of professional men?

Dr. Hancock's work is a very able and elaborate critical examination of the principal writings which have appeared at different times on the subject of epidemic and pestilential diseases; and we have no hesitation in saying, that its perusal will be very advantageous, in as much as it will help to prepare the way for more correct views and settled opinions on those important and much disputed points; though we are convinced it cannot have any operative influence on

* See page 585 of this number, where this paper should have come in, but was accidentally omitted.

the question of quarantine at the present time. The work is adapted for professional contemplation in the closet—not practical application to legislative enactments. As we have said before, an analytical delineation of the work would be absolutely impossible, without garbling and destroying the whole chain of arguments. The conclusions to which our author's researches and examinations of evidence have come, are shortly as follow.

"We have undoubted testimony, that certain chronic diseases, generally acknowledged to be contagious, originate amongst us in peculiar habits, under peculiar circumstances.

"We have also undoubted testimony, that of the acute diseases, common contagious fever originates at certain times, in peculiar situations.

"It is supported by equal authority, that common contagious fever may be so aggravated by peculiar circumstances, as to assume characters of extraordinary malignity.

"It is no less true, that the highest degree of common contagious fever has on some occasions approached so nearly to the plague, that a certain diagnosis has in vain been attempted.

"Therefore, the probability, indeed the most natural inference is, that the disease called Plague is only the highest degree or a variety of common contagious fever: and as the latter often originates without contagion, so it is inferred may the former; and if in one instance, so it may in many: and where there is a strong predisposition, it is natural to conclude, that morbid effluvia from the diseased to the sound must assist in propagating the mischief, by acting as a powerful exciting cause." 350.

Dr. Hancock has thrown into an appendix some keen criticisms on the writings of Sir B. Faulkener, Dr. Granville, and Mr. Tully. And we were gratified to find that this ingenious author has taken the same view of certain transactions at Noya and Corfu, as we had done, before we saw his book. We regret exceedingly our inability to go into the volume generally or partially; but Dr. Hancock must be convinced himself that an analysis is impracticable. Distrusting our own powers, we put this volume into the hands of three friends successively; all of them interested in the discussion. They all returned the work with this observation—that it contained a great deal of interesting research and legitimate deductions; but that no analysis could give a fair or satisfactory view either of the data or inferences. We shall, however, introduce a single extract which, besides containing matter of high importance, may convey some idea of the bearings of our author's reasonings and researches.

"It appears to be amply demonstrated, by repeated observation, that animal effluvia, condensed and stagnant in a confined air, from a number of persons crowded in a small space, and surrounded by their own filth, even without the morbid action of a febrile affection, acquire a high degree of virulence, and become deleterious, if not to those accustomed to such an air (from the influence of habit) yet to others recently exposed to it.

"But if this be the case, so that the very clothes of the former may convey a poison into the open air, what are we to conclude, when, to the circumstances above noticed, are superadded corrupt food, the influence of sickly seasons, and the morbid progeny of vitiated humours—animal effluvia secreted from the human body in a state of malignant febrile action? Is it credible, if the former position be allowed, that from such a combination of circumstances, miasmata, endowed with a most pestilential contagious power, will not be generated?

"'The most pernicious *infection*, next the Plague,' says Lord Bacon, 'is the *smell* of the jail where prisoners have been long and close and nastily kept;'—'which has some similitude with a man's body, and consists of human flesh or sweat putrefied.'

*Nat. Hist.* 914."

"As in the former case, which I conceive to be parallel to the often-quoted seizure of three hundred persons at the Oxford Assizes, in 1577, (where we do not learn that a propagation of the disease took place in a purer air) it may be presumed that the mischief would soon cease; so in the latter, we have nothing but the evidence of facts to build upon. And these alone can inform us how a more malignant poison maintains its existence when transplanted to another soil; if a contagion so produced be obedient to the laws of other *contagions;* whether it loses its power by frequent transmission or by lapse of time; whether it selects the victims more nearly resembling those from whom it sprung or finds others more susceptible; whether it changes its type by change of season; whether it chooses the marsh or the sandy soil, the wooded or the bare, the city or the country, the air of the mountains or of the plains.

"We know but little how the various kinds of contagion differ from each other, and in what various ways infection may take place; but it is an undoubted truth, that as there is in bad morals a tendency to taint our neighbour, so is there in almost all febrile diseases, where filth and vitiated customs and impure air prevail, a peculiar tendency to spread by a species of physical contamination. All the secretions and exhalations seem then to partake of a virulent activity which helps to propagate a similar disease to another, not yet perhaps ready to yield up his strength to the prevailing malady, till he receive a taint from his sick comrade. How else are we to explain the accounts of intermittent fever, of remittent and bilious fevers, of cholera, dysentery, &c. being considered by men of no mean authority at times positively contagious?

"Whether in such secretions and exhalations there be a contagion capable of producing its kind in one predisposed; or whether it be specifically distinct from other *contagions* in more qualities than its less fixable nature, or whether the skin, or lungs, or stomach, receive the first morbid change, or lastly, a more direct impression be made upon the sensorium by the olfactory nerves; it is not likely we shall be able readily to determine. The mode of infection may differ in different diseases. It may differ even in the same: for it is probable the stomach would receive the variolous poison as well as the lungs and skin. But it is very certain that contagion, if it may be so called, produced and disseminated in the manner above noticed, has such a distinct relation to, and dependence upon a certain state of air, that separate if possible the sick, the mortality will diminish; disperse the sound, the progress will be retarded if not arrested; let the diseased even mix with their fellow-creatures at a small distance who are more favourably situated, the latter will scarcely suffer harm, while the former begin to recover from the very hour of their removal.

"Now facts and observations like these are not adduced for visionary purposes; they are of practical and useful application.

"Shall we therefore be such devoted contagionists that our specific virus must, under all circumstances, propagate its kind; or so confirmed in the opposite opinion, that we can presume there will be no danger in approaching a comrade's couch, as in case of the diseases just mentioned, and inhaling his breath and offensive effluvia, when we ourselves may be on the threshold of a similar disorder?" 257.

The above extract contains sentiments to which we unconditionally subscribe, and which, indeed, we have long entertained, and disseminated in this Journal. We must now take leave of Dr. Hancock, while we tender him our professional respect and esteem.

3. *Fatal Internal Hæmorrhage.** Dr. Newman, of Berlin, relates a curious case of internal hæmorrhage, without apparent rupture of vessels. A robust woman, aged 35, was exposed to cold and wet, during menstruation. The catamenial discharge ceased suddenly, and was succeeded by violent pain in the abdomen, and, in a short time, vomiting. Next morning she was carried to the Charity, "pale as death, and *cold*—pulse scarcely perceptible—tongue and lips pale yellow—eyes dim—respiration anxious—whole abdomen greatly distended and painful—whole surface of the body *hot*—(here is a strange incongruity on the part either of Dr. Newman or his translator)—feculent vomiting—suppression of stools."

It was judged that the patient laboured under highly dangerous inflammation of the bowels, and the treatment was shaped accordingly. Dissection (she died at noon of that day) proved the diagnosis incorrect. On opening the abdomen, more than three pints of bloody serum escaped—and two pounds nine ounces of coagulum were found in the pelvis. There was no inflammation—nor could a vessel or vessels, of any size, be found ruptured. The point whence the blood had issued, must (Dr. N. thinks) have been either the diaphragm or the right ovarium. On both surfaces of the diaphragm, on the right side, there was a portion completely red, and crowded

* Hufeland's Journal, in Med. Repository, Oct. 1821, p. 332.

with blood-vessels. "The right ovarium was thoroughly converted into a mass resembling coagulated blood."

Without injection, in these cases, it is very difficult to discover the vessels whence a hæmorrhage issues, even when they are of considerable size; at the same time, there is no doubt but that great quantities of blood will sometimes be discharged from the mouths of capillaries, by a kind of exhalation, and without any apparent breach of texture. We have noticed this case chiefly for the purpose of keeping up the attention of our younger brethren to those important conversions and irregular actions which occasionally take place when the healthy functions of an organ are abruptly disturbed—and even when a long-established morbid process is rudely interfered with. Practitioners in this country are rather apt to despise these considerations.

4. *Tinctura Papaveris Somnif.** Dr. Duncan's work on the tincture of lettuce suggested to Dr. Wilson a trial of tincture of the papaver somniferum, as a substitute for laudanum. He used every part, except the root, taken at the proper time for making the extract, and dried in the shade. His modus is as follows:—Pulv. pap. somnifer. ℥iv. spiritus communis ℔j. digest. for eight days and filter. This medicine he introduced into his practice, under the same principles which govern the administration of tincture of opium, but in double doses. He found it in that proportion as good an anodyne as the foreign opium.

5. *Hydrocephalus.*† It has occurred to Sir Gilbert Blane that the distention of the head and bregma, in hydrocephalus, "is owing to a want of firmness and due resistence in the boney compages of the skull, which consequently yield to that effort of pressure with which the brain, in its growth, acts on the parietes." Reflections of this kind suggested to our author "that mechanical compression of the head might be of use in the cure of hydrocephalus." He accordingly made trial of it in the case of a child thirteen months old, who had a head of preternatural size almost from birth, and where the bregma was unusually large. The child was otherwise rachitic. Sir Gilbert directed the head to be swathed with a roller as tight as possible, short of producing pain or uneasiness. Leeches were applied to the temples, a purgative given every two days. "An immediate amendment took place, which continued so that all complaint was removed in less than three months, except the curvature of the spine." Sir Gilbert, afraid of the old complaint that "there is nothing new under the sun," has searched the records of medicine for parallel

* Dr. Wilson, in Dr. Chapman's Journal, No. 4.

† Sir Gilbert Blane, Med. and Phys. Journal, Oct. 1821.

or precedent, but has not found any. The discovery is therefore original. But Sir Gilbert Blane is too good a medical logician to attach any importance to a single case, even were that case most unequivocal. In the present instance, we do not see just grounds for attributing the cure to the bandage. The pressure of a roller, so slight as not to occasion "pain or uneasiness" in an infant's head, must be next to nothing; and where leeches and purgatives were used cotemporaneously, it is needless to say that no conclusion can be safely drawn respecting the share which the bandage might have produced. In a P. S. indeed, Sir Gilbert Blane has wisely limited the application of pressure to "those indications of predisposition consisting in a large head, a tardy closing of the bregma, broad pupils, and rachitic diathesis, or to causes in which the acute stage has been vanquished by evacuations and mercury, &c." Within such restrictions, we can have little objection to the application of a roller to the head, or hope of its producing any beneficial effects there.

P. S. When we had closed the above paper, we read a case in the Edinburgh Journal, of same date, which divides the honour of the discovery between Sir Gilbert Blane and Mr. Hood of Ayton, Berwickshire. Mr. H. tried pressure in precisely the circumstances under which Sir Gilbert recommends it, but without success—namely, after the febrile symptoms and squinting had subsided, the head continuing to increase in size. Mr. Hood now bandaged the head, but this measure brought on fits, and was therefore abandoned. An operation was afterwards performed, and six ounces of limpid fluid drawn off by means of a trocar; but the child died.

6. *Kinine, Cinchonine, Sulphate of Kinine.** Messrs. Pelletier and Caventon, have long been convinced that a great number of vegetable substances, possessing strong powers over the animal economy, owed their energy to a salifiable base; as the morphine in opium, the strychnine in nux vomica, the veratrine in helebore, &c. They therefore began to experiment on cinchona, with the view of discovering a salifiable base, if any. They soon found that the *cinchonine* or crystallizing principle of Garnil of Lisbon, was a substance of this kind united with an oily matter. They combined this substance with different acids, ascertaining the form, proportion, and properties of its salts, which all had a bitter, sub-aromatic flavour, proper to the pale bark, which was only used by Garnil. The cinchonine itself has this peculiar taste, but much less marked, owing to its high degree of solubility. Pursuing the examination of the the pale bark, Messrs. P. and C. determined also the nature of the other component substances, which, joined to the cinchonine, are as

* Revue Medicale for July and August 1821. Philadelphia Journal, August 1821.

follows:—1. Cinchonine united to the kinic acid. 2. A green oily matter. 3. Cinchonic red. 4. Red soluble colouring matter (tannin.) 5. Yellowing matter. 6. Kinate of lime. 7. Gum. 8. Starch. 9. Ligneous matter.

"If we enter into a consideration of these substances, the properties of which have been examined in detail and with care by the authors of the memoir, we shall see that the greater number, viz. the cinchonic red, the oily matter, the gum, the ligneous matter, and the starch, cannot possess any of the medicinal properties of the bark, of which they have not the taste: the greater number of them are even insoluble. The soluble colouring matter or the tannin cannot either be regarded as the active substance in the cinchona, for if the latter owed its virtues to any tanning matter, other barks, very rich in this principle, ought also to have the properties of the cinchona in proportionate energy according to the quantity of tanning matter they contain, but which is contrary to medical experience. There remains the kinate of lime, but this salt when pure has no bitter taste, and is besides insoluble in alcohol. The alcoholic tinctures of bark, therefore, do not contain it, and they are nevertheless febrifuge. Every thing then induces us to believe that the active principle of the bark is the cinchonine; consequently, it is to this substance and to its salts, that the attention of the physician and his therapeutical researches ought to be directed." *Philadelph. Journ. p.* 263.

Messrs. Pelletier and Caventon, in analizing the yellow bark, obtained a substance not crystallizable, and differing from cinchonine in physical and chemical properties. It was a salifiable base, its capacity for acids being different from cinchonine, and its salts more bitter. This substance has been denominated *kinine.*

The analysis of *red* bark presented an extraordinary fact—the simultaneous presence of the cinchonine and the kinine, and each in a greater quantity than is afforded by the pale and yellow bark. The red bark is therefore justly regarded as the best.

*Process employed by Mr. Pelletier for procuring the Cinchonine and Kinine.*

"Repeated alcoholic tinctures of the bark are first made, and by evaporation the alcoholic extract is obtained. It is in this extract that the cinchonine or the kinine is found, which exists in the Peruvian bark. To obtain the alkaline substance of a suitable purity, the extract is boiled in a certain quantity of water, to which has been added a few drops of hydrochloric (muriatic) acid: the liquor after cooling is filtered—then concentrated, and treated with an excess of magnesia—boiling it for a few minutes, the liquor is again suffered to cool, and then filtered. The precipitate received on the filter is composed of kinine, calcined magnesia, tannin, and cinchonic red. Wash the precipitate with cold water, dry it in a sand bath, then treat it with boiling alcohol, which dissolves the alkali, and leaves the magnesia and the tannin united to the colouring matter.

It remains but to evaporate the alcohol, to obtain the kinine of a superior purity.

"The alkali of the cinchona thus prepared is sometimes impure by an admixture of oily matter—to separate which and purify it, we must dissolve it anew in an acid largely diluted with water, filter again the liquor, and treat it for the last time with magnesia and alcohol, as has been already mentioned." 264.*

The sulphate of kinine has now been employed by a great number of French physicians in fevers of type, and with considerable uniformity of success. M. Double has administered the salt in doses of two grains, morning and evening, which have been sufficient to cause the fever to cease. To prevent a relapse, however, its use was continued some days longer. In other experiments made at the *Hopital de la Charité*, five grains have been given daily in simple quotidian fevers, and continued for eight days. From the very first dose the recurrence of the paroxysms was prevented.

Dr. Bally has reported nine cases of fever to the Royal Academy of Medicine, cured radically by the sulphate of quinine. It appeared to M. Bally, that ten grains given in five doses during the apyrexia, were sufficient to check at once the fever. In autumnal intermittents, however, he conceives that larger doses will be necessary. Two great and obvious advantages of the salt of bark over the powder are, the smallness of the dose, whereby the stomach is saved from nausea or oppression, and the freedom from astringency, it being rather indeed of an aperient than an astringent nature. The dose may be carried to 28 or 30 grains in the 24 hours, which would be equal to about three ounces of the powder. In pernicious fevers where it is desirable, during a short remission, to introduce a large quantity of the cinchona, this new preparation will be of incalculable value!

M. Duval, second naval physician at Brest, has sent up a memoir

* The following process for extracting cinchonin from cinchona is given by M. Badolier, in the An. de Chemie, vol xvii. p. 273. "A pound of yellow bark bruised, is to be boiled in three pints of a very dilute solution of caustic potash. After the ebullition has continued a quarter of an hour, the liquor is to be suffered to cool, and strained through a fine cloth with pressure; the residuum is to be repeatedly washed and pressed. The cinchona thus washed is to be slightly heated in a sufficient quantity of water, adding gradually muriatic acid until litmus paper is slightly reddened, and stirring the mixture. When the liquor is near the boiling point, it is to be strained, and the cinchona strongly pressed; then add to the strained liquor, while hot, an ounce of sulphate of magnesia. After this, precipitate the whole with caustic potash slightly in excess. When the liquor is cold, the precipitate is to be collected on a filter, washed, and dryed, then treated with alcohol, as directed by M. M. Pelletier and Caventon, in order to obtain the cinchonin. When sulphuric acid is added to the cinchonin immediately after the separation of the alcohol crystals of sulphate of cinchonin are obtained, which, when washed with a little water, are of a very fine white colour."

of seventeen cases of intermittent fevers of different types, cured by the sulphate of kinine. The medicine was prepared by Messrs. Vasse and Colomb, of Brest, who employed a new process, which consisted in boiling the yellow bark in water acidulated with acetic acid, and precipitating the alcaline principle by ammonia. M. Renauldin, M. Hallé, and M. Roubiquet have also furnished various notices of this interesting discovery. We trust that what we have here adduced will stimulate our chemists—particularly the *Hall*, to furnish the English practitioners with a medicine which promises to be a very valuable addition to the materia medica.

7. *Epistaxis.** Hæmorrhages are formidable occurrences at all times, and in all places. The application of cold in hæmorrhage from the nose and fauces especially, is no new principle, but it is generally done in a partial or local manner. Dr. Platt having a severe case of epistaxis, in which four pounds of blood were computed to be lost, and the bleeding persisting in spite of all the usual remedies, he directed, in consultation with another physician, Dr. Moorers, the patient to be immersed in a bath of well water, till rigor was induced. This effect took place in about a minute after the immersion, and continued a few minutes after coming out of the water. The hæmorrhage abated before he left the bath, soon ceased entirely, and did not afterwards return. Such is the general sympathy between the capillaries of the skin and those of all other parts of the system, that we believe cold might be more freely used to the whole external surface, in internal hæmorrhages than it now is. Dr. Bond, of Philadelphia, was in the habit of using the cold bath with success, even in pulmonary hæmorrhage.

8. *Prussic Acid.*† Dr. M'Leod, Physician to the Westminster General Dispensary, has given an extensive trial to the prussic acid in pectoral complaints, but has discontinued its exhibition, "because he was unable to discover any certain indication which it was capable of fulfilling." He did not find any deleterious effects from the remedy, except once, and that was not productive of serious inconvenience. In complaints of a spasmodic nature, such as hooping-cough, Dr. M'Leod believes this acid may be of use—"but the only class of cases in which he feels any real confidence in its exhibition, is dyspepsia, attended with much pain in the stomach, or anomalous feelings about the chest and heart. Indeed he has known it afford relief in structural affections of this organ." A new property, however, of the prussic acid is here developed—namely, the

* Dr. Platt, in Dr. Chapman's Journal, No. 4.

† Dr. M'Leod. Med. and Phys. Journ. 272.

power of ulcerating the gums and producing ptyalism. Dr. Granville appends some remarks to this communication of Dr. M'Leod, and quotes three cases, related by Dr. Nancrede of Philadelphia, in which the said acid appeared to produce considerable benefit in pectoral complaints. We need only add, that Dr. M'Leod's experience confirms the observations of Dr. Elliotson, and also of the reviewer of Dr. Elliotson's work in our 4th number for March last.

9. *Partial Retinal Paralysis.** 1. Læcour, a soldier in the Royal Guard, received, 19th November, 1820, a thrust of a sharp foil between the globe of the right eye, (which was not touched) and the internal paries of the orbit, the weapon penetrating, by all accounts, three inches or thereabouts. The wound healed, but Læcour, when looking with the right eye alone, could only see the perpendicular half of objects placed in the antero-posterior axis of that eye. As it was with that half of the eye next to the nose, which Læcour saw objects, so when these objects were carried towards the left of the patient, he could see them entire; while, if carried in a contrary direction, he lost sight of them, *in toto*, at a time when they could be distinctly seen with the left eye, if open. Thus it appeared, that the inner or nasal half of the retina was paralytic. In this state he was seen by the members of the *Faculté de Medecine*, three months after the accident occurred. A few weeks subsequently, Læcour committed some excesses, was seized with phrenitis and enteritis at the same time, and died on the fourth day of the disease.

*Necrologic Examination.* Invaginations of the small intestines and peritoneal inflammation were observed in the abdomen, but the head was the greatest object of research, on account of the retinal paralysis before described. It was found that the point of the foil had pierced the orbital plate, and grooved the under surface of the anterior lobe of the right hemisphere, passing obliquely behind the point of the falx, and above the decussation of the optic nerves, stopping under the inferior paries of the left lateral ventricle. This tract was marked by a kind of reddish clot of fibrinous substance, but without any appearance of suppuration. Immediately surrounding this clot, the cerebral substance was yellow, and manifestly altered in consistence to about half a line in depth. Some slight serosity was effused under the left hemisphere.

Another, somewhat similar case, is transcribed from Demours. Madame de Pompadour took cold in December 1762, in the Park of Versailles, and awoke the succeeding morning capable of seeing only the half of objects with the left eye. If a person stood right before and close to her, she could not see his right cheek or corresponding side of the nose. The iris preserved only half its mo-

* Two cases of paralysis of half of the retina (amaurosis dimidiata) par le Baron Larrey. Revue Medicale, August 1821.

tions of dilatation and contraction. This affection disappeared in about two months by means of stimulants to the skin in the neighbourhood of the eye.

10. *Inverted Toe-Nail.** This troublesome little complaint is not beneath the notice of the surgeon. Dr. Meigs thinks it probable that the disease is generally produced by the practice of cutting at the lateral edge of the nail, with a sharp-pointed pen-knife. In this process, if the skin be once abraided, a sore is formed, which is perpetually irritated by the pressure of the nail above, till fungous granulations shoot out. As the nail does not yield its position, it appears, by the swelling of the soft parts on one or both sides, to have grown down into the flesh, while, in reality, the edge is no deeper than is natural. After reducing inflammation by the usual means, our author has cured the complaint by the following simple process:—

" Let a small pledget of lint, just large enough to cover all the granulations, and of sufficient thickness to act as a compress, be neatly adjusted, over which a roller of linen three quarters of an inch wide, and eight or ten inches long, is to be applied, having one end previously spread with adhesive plaster. By this method we are enabled, with great ease, to make it act not only on the compress, which will destroy the granulations very rapidly, but, by confining the toe and nail, to prevent even the *small* degree of sliding motion or friction formerly mentioned, thus doing away one principal cause of the disease.

" By pursuing this treatment, the patient will generally recover, even while walking about—and the pain certainly is removed very quickly, for he can now wear a shoe, who before found a tight stocking incommodious." *Philadelphia Journal, p.* 266.

11. *Extract of Opium.*† Majendie thinks that the variable effects of opium on the human frame are owing, in all likelihood, to the opposite principles of which the opium is composed. It is considered therefore, that, as those who take morphium do not experience the exciting properties resulting from the aqueous extract of the shops, the process proposed by M. Robiquet should be adopted by apothecaries and chemists, since it well accomplishes the object in view—the separation of narcotine.

M. Robiquet macerates opium, cut into small slices, in cold water, and evaporates the filtered solution to the consistence of thick syrup. This extract is to be repeatedly agitated with ether in a proper vessel.

* Dr. Meigs. Chapman's Philadelphia Journal, No. 4.

† Journal de Pharmacie, Mai 1821.

The ethereal tincture is then decanted, and submitted to distillation, in order to separate the ether. This operation is repeated as long as crystals of narcotine are obtained. When the ether no longer acts, the solution of opium is evaporated, and the extract prepared. As the same ether may be employed again in the preparation of extract, the process is not so expensive as might be imagined at first sight. It is said that excellent effects have already resulted from the exhibition of this preparation.

12. *Nitrate of Silver.** The *Giornale di Fisica*, tom. xi, contains, at p. 355, a paper by Il C. Sementini, on the use of nitrate of silver in cases of epilepsy. After remarking on the difficulty which occurs in treating such cases, and the good effects which have been observed in using the nitrate of silver, and its superiority in this respect over all other remedies, both as to the effect it produces, and the little inconvenience it causes; the Cavalier states, that to secure the good effects belonging to it, the nitrate of silver should be well triturated with the vegetable extract, in combination with which it is given; that the first doses should be small, and the quantity gradually increased to six or eight grains, or even more, in a day: that the use should not be continued very long together; and that the patient should keep out of the action of light. The latter precaution is necessary, to prevent the discolouration of the skin, which sometimes happens after a long and copious use of this remedy. The precaution, however, only regards avoiding the meridian sun-light.

"It frequently happens, in the use of this medicine, that a species of cutaneous eruption, consisting of small pustules, occurs. This may be regarded as a certain proof of the good effects of the medicine.

"In the early part of this paper, Il C. Sementini, in endeavouring to remove the impression existing against nitrate of silver, because of its poisonous qualities, remarks, that being mixed with vegetable extract, it is not really the salt, but the oxide, that is given; and, therefore, the observations of M. Orfila, on the nitrate as a poison, have nothing to do with the power of the remedy. At the same time, as an argument for using the nitrate in place of the oxide, it is remarked, that at the moment of decomposition a combination is, probably, effected between the extract and the oxide; and that actually the salt is found most efficacious.

"Being assured of the use of nitrate of silver in epileptic affections, and reasoning upon its tonic effect, Il C. Sementini was induced to try its powers as a remedy in cases of paralysis. The first instance quoted is of a gilder, who, probably from the fumes of mercury, had become very paralytic. An eighth of a grain of nitrate of silver was prescribed at first, but the dose was increased every

* Gior. di Fisica. See also Med. Repos. No. 95.

other day: by the time that three grains were taken the good effects were evident, and in twenty days more the man was perfectly restored. In another instance, every part of the body and limbs were paralyzed but the head. A small quantity was given at first, but it was increased to eight grains per day, and it effected a cure.

"Three other instances are then adduced, in all of which cures were effected: and the Cavalier expresses his hopes, that, in the hands of other medical men, it will be found as effective and as important as in his own." *Med. Repos. p.* 434.

We have, within these eighteen months, checked epileptic paroxysms in three patients, by nitrate of silver, carried to five grains daily, but not continued longer than two months at one time. We observed no other effect than, first, prolongation of the intervals, and afterwards cessation of the paroxysms.

13. *Arachnitis, Cerebritis, Enteritis.** Picard, a female 50 years of age, in a state of tranquil dementia, after repeated attacks of paralysis, was seized, on the 7th January, with insensibility, followed by coma. On the 8th, great alteration of the features—strabismus—stupid look—insensibility of the iris—spasmodic contraction of the facial muscles, equally on both sides—mobility of the upper and lower members—pulse hard, frequent, irregular—respiration laborious—tongue yellow, dry, red at the edges—thirst intense—stools involuntary—the patient neither understands nor feels any thing. In the evening a convulsive paroxysm, during which she got up and fell down several times. On the 9th, 10th, 11th, and 12th January, nearly the same symptoms—the evening paroxysms being still very violent. 13th. The lower extremities, hitherto moveable, became stiff, paralytic, and insensible—the teeth and tongue covered with a fuliginous substance. 16th. The right arm is paralytic—in the evening the left arm in the same state—all the symptoms aggravated. In this deplorable state she continued three days, and then expired.

*Dissection.* Dura mater strongly adherent to the cranium—tunica arachnoidea raised from the pia mater by a stratum of reddish serosity—the arachnoid itself, throughout its whole extent, thickened and red. The vessels of the pia mater gorged with blood, and not to be detached from the cortical substance beneath. In the substance of the left hemisphere there was a disorganized portion, of a pultaceous, yellow, whitish appearance, almost liquid. This disorganization penetrated to the bottom of the posterior lobe—the middle and anterior were sound. In the right hemisphere there was a nearly similar disorganization. The corpus stratum of this side presented, at its upper portion, a yellowish cicatrix, lined by a firm

* Pinel fils; Revue Medicale, Juillet 1821. We request the reader's attention to this case, and the subsequent reflections.

membrane, and containing a small brown inorganized substance—doubtless the residuum of an old cerebral hæmorrhage. The cerebellum and spinal marrow were sound.

The heart large—the parietes of the left ventricle an inch in thickness, and the cavity very much diminished—aortic orifice cartilaginous, and the valves ossified. The liver covered with an albuminous exudation. The mucous membrane of the intestinal canal, including the stomach, highly inflamed.

*Reflections of M. Pinel.* In this case we see three severe inflammations nearly simultaneous—namely, inflammation of the arachnoid; of the brain itself; and of the mucous lining of the alimentary canal. The distinctive symptoms which denoted them during life, were, for the *arachnitis*, the stupor, the somnolency, the contraction of the fascial muscles, without any paralysis. For the *cerebritis*, we had the paralytic affection, first, of the right and afterwards of the left side, indicating an alteration successively in the left and right hemispheres of the brain. For the *muco-enteritis** we have the coating, first yellow and then fuliginous, of the tongue and teeth—and the intense thirst. As symptoms common to all the three inflammations, we have the great alteration in the features; the hardness, frequency, and irregularity of the pulse; the laborious respiration; and the involuntary stools.

The comparative march of the encephalic inflammations is remarkable. The invasion of the arachnitis is sudden, and its symptoms remain stationary during the first four days. On the fifth day a new phenomenon presents itself—a paralytic rigidity (roideur paralytique) of the inferior extremities, announcing an affection of the cerebral pulp itself, particularly towards its superior portion. On the ninth day the immobility of the *right* arm indicated a deeper lesion of the *left* hemisphere—the paralysis gaining the *left* arm evinced the extension of the disease to the *right* hemisphere—in short, by an attentive examination of the symptoms and their progressive march, one could follow, as it were, with the eye, the encephalic alteration, at first confined to the meninges, soon afterwards attacking the cortical substance, and finally penetrating profoundly into the hemispheres of the brain.

There is every reason to attribute the laborious respiration to the hypertrophy of the heart, as also the congestion, or, as it were, stagnation (stase du sang) of blood in the capillaries of the mucous mem-

---

* We have no term to distinguish inflammation of the mucous lining from that of the peritoneal covering of the alimentary canal, though the one is a mucous and the other a serous membrane. Surely we should have designating terms for these very different kinds of inflammation. We think there can be no impropriety, in proposing "muco-gastritis" and "muco-enteritis," to imply inflammation of the interior tunics of the stomach and bowels; while sero-gastritis or peritoneo-gastritis—and sero-enteritis or peritoneo-enteritis, shall designate phlogosis of the outer covering of the viscera in question.—*Rev.*

brane of the stomach and intestines, ending in decided phlogosis of that membrane. This effect, M. Pinel justly observes, is a very frequent one consequent on diseases of the heart.*

M. Pinel, who has the range of the Salpetriere for observation, relates three other important cases, for which we have not room in this number of our Review. Our readers are all aware that we are no advocates for useless refinements and hair-breadth distinctions; but we believe there are many men in our profession, high in rank, and consequently powerful in influence or example, who set their faces against all such investigations as M. Pinel and many others are now pursuing. But we shall not cease to remonstrate and rebel against these Goths and Vandals of medical science, who would stifle in embryo all attempts at clearing away some of the many doubts and uncertainties which beset us on all sides, and draw on us the appellation of *conjectural* actors. If we do not advance, and that with vigour, we must retrograde. But we hope there is too much zeal afloat, and too much intellect in operation among us, to allow of our pausing in the arduous and praiseworthy spirit of investigation which characterizes the age we live in.

14. *Inflammation of the Heart fatal in ten hours.*† A man, 29 years of age, presented himself to Dr. Ulrich, complaining of a tightness of the chest, and feeling as if flatus mounted up into the thorax. At the same time, he had palpitation of the heart. As he was a man of very sedentary habits, often sitting up whole nights in

---

* M. Pinel here makes a short physiological digression, in which he expresses a conviction that, after all the experiments on the circulation, the heart is the main, if not the only, organ, which moves the blood under ordinary circumstances. Pathology, he thinks, has here thrown light on physiology. We shall quote the concluding passage in the author's own words.

"Nous voyons, en effet, que le sang stagne dans les capillaires, soit lorsque la circulation est trop active, comme dans les hypertrophies des ventricules du cœur avec agrandissement de leurs cavités; soit lorsque, ralenti dans son cours, le sang n'a pas assez de force pour parvenir jusqu'aux extrémités artérielles, comme on l'observe dans les hypertrophies des ventricules avec rétrécissement de leurs cavités. Or, que la circulation soit trop active ou ne le soit pas assez, il y a stase du sang. N'est-ill pas évident alors que le cœur est l'organe essentiel et primitif de la circulation capillaire, puisque s'il envoie trop de sang aux extrémités artérielles, l'accumulation du fluide en comprime la masse et empêche sa circulation, et que s'il n'en envoie pas assez, le fluide ne reçoit pas une impulsion suffisante à sa circulation pleine et entière: d'où il résulte stagnation du sang dans les deux cas. Et si le cœur est en effet le principal agent de la circulation capillaire, il doit nécessairement avoir aussi une grande influence sur le mouvement du sang veineux." *Revue Medicale*, p. 310.

† Dr. Ulrich; Journ. Comp. des Sciences Medicales. Sept. 1821.

study, Dr. Ulrich recommended him to take exercise, and prescribed some pills to keep the bowels free. Two months afterwards, Dr. U. observed that his countenance was become yellow, and he now complained of an indescribable sensation in the chest, but had no pain, nor was his health perceptibly altered. He was a man of eccentric character, but possessing much talent and genius. On the 6th October, 1820, at five o'clock in the afternoon, he came to Dr. U. begging relief for most violent pains which he experienced in his chest—pains which were of quite a different nature, he observed, from those strange sensations he before alluded to. He had eaten his dinner, with appetite, at three o'clock, and the pains had progressively increased from that time till five. He could assign no cause, unless it was that of having washed his chest with cold water in the morning. The heart did not beat very inordinately—the pulse was soft, and not more frequent than in health. The pain extended from the region of the heart throughout the chest. The skin was cool, nor was there any appearance of pyrexia. The abdomen was soft and void of tenderness on pressure. The patient had had a stool in the morning. His countenance was not expressive of any anxiety. He conversed calmy on the complaint, and asked Dr. U. if he did not think that blood-letting would be proper. The disease appeared to Dr. Ulrich to be spasmodic rather than inflammatory, and therefore he determined to admintster a gentle diaphoretic, and wait the further development of the complaint. About eight o'clock in the evening, Dr. U. called on the patient, and found him pacing his room, still complaining of considerable pains, although they were somewhat mitigated. The physician and patient again conversed on indifferent subjects. The pulse, action of the heart, temperature, and countenance, were the same as three hours before. Even now Dr. Ulrich had no suspicion of cardiac inflammation, and took leave of the patient in full persuasion of finding him better next morning.

At midnight, however, he was summoned, in consequence of the pains having augmented and become almost insupportable. Dr. U. found the patient on his bed, extremely restless, and shifting his position every moment—talking very fast—sometimes crying out loudly, and affirming that he must soon expire if not relieved. His countenance and figure now expressed great anxiety—he frequently threw back his head—respired quickly—felt the pain much augmented when any pressure was made on the chest—but had no cough.

Dr. U. now became impressed with the idea of carditis, and began to think of blood-letting; but as the pulse continued soft and quiet—as the hands and feet were warm—as he had no syncope—and as the patient assured Dr. U. that the action of the heart was not greater than natural—finally, as *pain* was a very equivocal symptom of inflammation, (" the patient appeared phrenitic, jumping out of bed, and rolling himself on the floor,") he determined to defer the blood-letting until morning. He prescribed twelve grains of nitre with half a grain of the aqueous extract of opium every hour. While the medicine was preparing at the apothecary's, the patient's state became so much worse that Dr. U. saw the business was up, and that neither bleeding nor any other measure would save him. The respiration became stertorous—the extremities cold—the pulse extinct—forehead covered with cold sweat—and death closed the scene in ten minutes after he swallowed the first powder—ten hours from the apparent invasion of the disease, and seven after Dr. U. was consulted. Dr. U. on enquiry, found that this gentleman was greatly embarrassed in his circumstances, and very unhappy in his mind for some time past; and on reviewing the history and termination of the case, he became convinced that disease of the heart had existed long before the present time; but that acute inflammation had probably lately supervened. This is what Dr. U. now tells us. But we may remark, *en passant*, that some men make wonderfully acute *prognoses* in the cases they relate, *after* dissection has set things in a proper point of view. If such was Dr. Ulrich's natural sagacity, we lament that he did not exercise it, except in the short period between death and dissection.

*Necrotomy.* Heart not adherent to the pericardium. About six ounces of clear yellow liquid in the cavity of the pericardium. The heart appeared somewhat more voluminous than natural, but this was owing to the state of the large vessels that rose from its cavities. The anterior surface of the heart was unequivocally inflamed, with large red spots, and abundance of coagulable lymph scattered around it. The exterior of the root of the aorta was red, and covered with a slight layer of coagulable lymph. It was enlarged to nearly double its calibre, and several aneurismal tumours projected from its sides, but all communicating with the canal of the artery. Two of these pressed upon the under surface of the heart, and gave it the appearance of being amplified in volume. The interior of the root and arch of the aorta was of a crimson colour. There were some other morbid appearances about the heart, but not worth notice. There was some effusion in both sides of the chest, particularly the right side, where it amounted to thirteen ounces.

We think the above case may prove an instructive lesson to our junior brethren—nay, to some of those whose heads are ready to crack with experience, but who, some how or other, do occasionally contrive to get on the wrong side of a case, in spite of all their boasted *tact*. From the days of Hippocrates the pulse has been pronounced the most fallacious guide, and yet it every day misleads practitioners. No one diagnostic mark can be trusted to in diseases. We must look to the whole tenor of the patient's feelings and functions—and if there is much suffering, we ought to suspect that some important organ is labouring, though silently, and ought to be relieved if possible, or guarded from danger by those well known means which, even if wrongly used, cannot do much harm to spasm —but, if neglected, bring ruin on the part where inflammation is ravaging unseen.

15. *Sarcocele.* M. Maunoir of Geneva has recently recommended to the surgical profession a mode of treating sarcocele, without extirpation of the testicle. This consists in cutting off the main current of blood from the organ, by tying the spermatic arteries. He recommends these vessels to be tied close up to the ring, so as to interrupt the circulation through as many of the small branches as possible. Previous to an operation, however, it is necessary to distinguish sarcocele from medullary fungus of the testicle, with which, he thinks, it is too often confounded.

"In sarcocele," says he, "the tumour is generally more uniform, and more firm; its volume does not exceed twice or thrice that of the healthy testicle; the spermatic cord remains unaffected. In the medullary fungus of the testicle, the organ usually acquires a more considerable size; the body of the testicle and the epididymis becomes soft, and affords a deceptive sensation of fluctuation, which has sometimes caused the case to be mistaken for hydrocele. If the disease is far advanced, the scrotum changes colour, becomes livid, and at length ulcerates. When this happens, the chord commonly participates in the tumefaction, and in the deceptive fluctuation above mentioned. The swelling of the chord continues even up to the abdominal ring, and the abdomen cannot be pressed without causing great pain in the loins." *Foreign Journal, p.* 560.

M. Maunoir put this proposal to the test in two cases. In one, the spermatic artery was tied on the 8th of June, 1820, and divided below the ligature. By the 4th July, an evident diminution had taken place in the tumour. On the 20th August the patient quitted the hospital, his testicle being without pain and not larger than the sound one. Hydrocele was combined with enlargement of the testicle in this case, and the water had been twelve times evacuated, but still returned. The pain ceased the moment the artery was divided, and the hydrocele was radically cured. The suggestion deserves some attention from our surgical brethren.

16. *Dislocation of the Patella.** *September* 1821. "A coal heaver fell down so as to allow both the fore and hinder wheels of an empty coal-waggon to pass over his right knee, in a direction from the inner to the outer side of the joint. He was immediately brought to St. Bartholomew Hospital. The patella rested perpendicularly on its internal edge, and its external edge was inclined directly forwards, so that its upper surface was turned inwards, and its under or articular surface outwards; in the same way as the extended hand might rest on its ulnar edge, instead of its palm. I speak of the horizontal position of the body. The bone was placed so nearly in a perpendicular direction, that it was not easy at first to ascertain which was its upper, and which its under or articular surface, but this was distinguished by comparison with the superior surface of

* Mr. Wheeler, Quarterly Journal of Foreign Medicine, No. 12.

the opposite patella. The insertion of the vastus externus into the external edge was the only tendinous attachment which was much stretched. I reduced the dislocation suddenly, but with some difficulty, by bending the thigh very much on the pelvis, drawing downwards the flexor muscles as much as possible, and by forcibly raising the bone at the same time that I turned it in its natural direction. Some inflammation in the joint followed, but was arrested by the usual means." 563.

The following case related by M. Combette, in the Journal Generale de Medecine, may be here introduced with propriety.

M. C. thirty years old, dislocated his patella in wrestling by a motion which he could not describe. There was a projection of the knee; the leg was permanently extended. The patella rested completely on its edge, its articular surface being turned outwards. The internal half of its circumference was fixed in the articular groove formed by the elevation of the sides of the anterior part of the condyles of the femur.

" Reduction.—An assistant raised the thigh, and with one hand M. Combette bent the leg on the thigh, while, with the other, he turned the patella from within outwards. The reduction was effected in an instant, and was followed by slight swelling and weakness. Six months afterwards, in dancing, the same accident happened in a less complete degree; for the patient was able to replace the bone himself. The joint is perfectly well formed." 563.

17. *Arteritis and Phlebitis.* Dr. S. Sprengel relates the following curious and interesting case of inflammation of veins and arteries from a wound, in the *Journal Complementaire des Sciences Medicales*:—"A young man, lately embarked in the military service, was sent to the hospital for a wound in the right hand, which he said was accidently produced by a hatchet, but which was very evidently the result of design. Be that as it may, the wound was properly dressed, and every thing went on well for the first two days; but in the evening of the second day, a violent symptomatic fever was kindled, which, though mitigated for a time by a copious bleeding, came on again with more intensity than ever, accompanied by gastric symptoms. An emetic relieved the latter. The wound now suppurated kindly, and on the fifth day there was every appearance of a speedy cure. His companions at this time persuaded him that he would be severely punished for the attempt at maiming himself, which, joined to the chagrin of being disappointed in his hopes of discharge from the military service, threw him into a low nervous fever, while an erysipelatous inflammation began to spread over the back of the wounded hand, and from that up the arm, in the direction of the great vessels. The fever now assumed the typhoid form, although the wound itself preserved a healthy appearance, and never ceased to discharge good pus. An abscess formed on the wrist, and was opened, when a great quantity of sero-sanguineous pus was discharged. On the 18th November a great hæmorrhage took place from the wound, without any apparent cause. This was soon stopped. The process of suppuration was now arrested, and the wound looked dry and shrivelled. The patient died on the 20th November, eighteen days from the infliction of the wound.

*Dissection.* Nothing remarkable in any of the splanchnic cavities. Sinuses and abscesses were found along the arm. The sheath of the radial nerve was a little inflamed, the radial and ulnar arteries, from the wrist to the middle of the fore arm being filled with pus. The lining coat of these vessels was thickened, corroded, and covered with coagulable lymph. The brachial, axillary, and subclavian arteries were sound. The veins, on the lower part of the fore-arm, presented the same phlogosed appearances as the arteries. On the clavicle an abscess was found filled with ichorous pus, the bone being denuded of its periosteum, and carious. Yet there was no affection of the vessels in the vicinity of this abscess. No other morbid appearance was found in any part of the body."

The above case offers a good example of the effects of moral emotions over the physical structure of our frames.

18. *Ruptured Perineum, &c. in Parturition.** This is a very aukward accident, and will sometimes occur where the ablest accoucheurs are employed. In our own practice, these ruptures always happened where the perineum was well supported, and we never recollect seeing an instance of it where women were delivered before the arrival of the doctor, (no uncommon incident,) and consequently where the perineum was left to its fate. We do not therefore agree with M. Montain, that the accident is invariably owing to neglect in the accoucheur. Some authors employ the suture, others leave the process of reparation to Nature. But to the case before us. The labour had been a laborious one, and turning was necessary. A month after delivery our author found the woman under fever, with a fetid discharge from the vagina, the latter being found filled with *fæces*, the fourchet, perineum, sphincter, and rectum torn, and forming a great and terrible wound, confounding the two passages. The wound presented no appearances of commencing cicatrization. Suture was therefore determined on, and the needle passed pretty deep, probably includiug a portion of vagina. One stitch only was employed, and was found sufficient to keep the sides of the wound in contact for a considerable space. To facilitate the discharge from both passages, an elastic gum tube was placed in each, and the thighs brought together, while the patient was ordered to lie on her side. All discharges were carefully washed from the parts, and an amend-

* Revue Medicale, M. Montain.

ment soon appeared. The fever subsided, and on the 8th day the compresses were removed. Two days afterwards the ligature was also removed, the cicatrization being complete. In a month the patient was considered as perfectly well.

19. *A Sketch of Congestive Fever—drawn nearly two centuries ago.*

" Sæpe vero medici decipiuntur a putridarum febrium miti ac leni calore in cute observato. Etenim cum natura opprimitur ac fere humorum multitudine suffocatur, quo color nativus alioquin ardens, sese exinde intus contineat, nec foras moveatur, partes corporis exteriores parum calent. Has febres aliqui fumantes magis quam suffocantes appellant, quod in ipsis non flamma, seu ardor calorve, foras per cutis meatus moveatur, sed fumida potius neque ita urens exhalatio, seu fumus, uti in igne lignorum viridium vel temere congestorum copia obruto, et suffocato pro flamma fumum excitari observamus, aut ubi ita natura deficit, ut nisi ex putrefactis succis calor accensus non multus sit; neque ad cutem copiose perveniat."—*Prosper Alpinus, de Præsagienda Vita et Morte Ægrot. Cap.* xiv.

" Repente mutari ad frigidum corpora, nisi accessione id fiat; semper malum:—denique maligni humoris viscus nobile lacessentis, vel vehementis inflammationis actione intus totum collectum esse significat:"—' Et hinc est quod in morbo summe maligno hujusmodi mutationes, Galen in Prorrhet. dixit esse lethales, easque extinctione naturalis caliditatis fieri.' Pulsus summe inæqualis, prælanquidique contracti. Resolutum conjicimus, si prius resolutionis aliqua causa præcesserit, uti febris vehemens, assidua, vigiliæ item assiduæ dolores ingentes, immodicæque vacuones cum sanguinis tum humorum."—*Prosp. A. Ch.* xvi.

What Prosper Alpinus faintly sketched, Drs. Jackson and Armstrong have now distinctly painted. *P.*

---

From the great length of several of the analytical articles in this number of the Journal, our PERISCOPE has been somewhat less copious than we intended, notwithstanding the exertion which we have made in compressing the matter and setting it in small type. In this, as in every other number of the Journal, (except No. 1,) we have considerably exceeded the regular limits of the work—often at the extra expence of ten or fifteen pounds sterling. The liberal, we might perhaps say, unprecedented encouragement which the Journal has experienced from the professional public, demands a suitable return on ours; and we venture to affirm, that no labour or expense will be spared in rendering the work equivalent to the object for which it was instituted—the more uniform diffusion of professional information through all classes of medical society.

